# A Comparison of Patients’ Local Conceptions of Illness and Medicines in the Context of C-Reactive Protein Biomarker Testing in Chiang Rai and Yangon

**DOI:** 10.4269/ajtmh.17-0906

**Published:** 2018-04-09

**Authors:** Yuzana Khine Zaw, Nutcha Charoenboon, Marco J. Haenssgen, Yoel Lubell

**Affiliations:** 1 Centre for Tropical Medicine and Global Health, Nuffield Department of Clinical Medicine, University of Oxford, Oxford, United Kingdom;; 2 Mahidol Oxford Tropical Medicine Research Unit (MORU), Faculty of Tropical Medicine, Mahidol University, Bangkok, Thailand;; 3 Department of Global Health and Development, London School of Hygiene & Tropical Medicine, London, United Kingdom;; 4 CABDyN Complexity Centre, Saïd Business School, University of Oxford, Oxford, United Kingdom;; 5 Green Templeton College, United Kingdom

## Abstract

Antibiotic resistance is not solely a medical but also a social problem, influenced partly by patients’ treatment-seeking behavior and their conceptions of illness and medicines. Situated within the context of a clinical trial of C-reactive protein (CRP) biomarker testing to reduce antibiotic over-prescription at the primary care level, our study explores and compares the narratives of 58 fever patients in Chiang Rai (Thailand) and Yangon (Myanmar). Our objectives are to 1) compare local conceptions of illness and medicines in relation to health-care seeking and antibiotic demand; and to 2) understand how these conceptions could influence CRP point-of-care testing (POCT) at the primary care level in low- and middle-income country settings. We thereby go beyond the current knowledge about antimicrobial resistance and CRP POCT, which consists primarily of clinical research and quantitative data. We find that CRP POCT in Chiang Rai and Yangon interacted with fever patients’ preexisting conceptions of illness and medicines, their treatment-seeking behavior, and their health-care experiences, which has led to new interpretations of the test, potentially unforeseen exclusion patterns, implications for patients’ self-assessed illness severity, and an increase in the status of the formal health-care facilities that provide the test. Although we expected that local conceptions of illness diverge from inbuilt assumptions of clinical interventions, we conclude that this mismatch can undermine the intervention and potentially reproduce problematic equity patterns among CRP POCT users and nonusers. As a partial solution, implementers may consider applying the test after clinical examination to validate rather than direct prescription processes.

## INTRODUCTION

Antimicrobials have been considered the basis of modern medicine and are one of the most frequently purchased drug types globally, but their effectiveness is challenged by growing antimicrobial resistance (AMR).^1–3^ Not only high-income but also low- and middle-income countries (LMICs) are affected by AMR because it can compromise the management of leading causes of death such as gastrointestinal, respiratory, sexually transmitted, and nosocomial infections.^4^ Several national governments and international organizations have thus declared AMR as a global health emergency.^3^

This article addresses AMR from a social perspective, focusing specifically on patients’ antibiotic use against the backdrop of antibiotic resistance (ABR; a subset of AMR). We know from the anthropological literature that patients’ conceptions of illness and medicines influence their health-care seeking and medicine use. Yet, our understanding of the social dimensions of ABR remains surprisingly narrow—indicated by the persistent narrow focus of national and global AMR policies on awareness raising (rather than e.g., socioeconomic drivers of resistance such as poverty), by repeated calls for more social research into AMR in general^5–7^ and by a social science share of only 0.53% out of 17,675 documents with the key words “AMR” according to the *Scopus* database (as of July 31, 2017).^8^ The social research gap is problematic because it affects how we think about the increasing global efforts to curb AMR.

Among these efforts are calls for rapid diagnostic tests (RDTs) such as C-reactive protein (CRP; a blood test marker for inflammation) point-of-care testing (POCT) to inform health-care workers’ (HCWs) antibiotic prescription decisions in LMICs.^9–11^ By indicating whether infections have bacterial or nonbacterial causes, clinical research compares the potential role of CRP POCT to malaria RDTs, which can help reduce patient uptake of unnecessary antimalarial treatment.^12,13^ However, the anthropological literature on malaria RDTs has cautioned repeatedly that local conceptions of malaria, social relationships, and treatment-seeking behavior can influence test effectiveness, stimulate heterogenous forms of use, and entail unforeseen and potentially adverse consequences for the users.^14–16^ C-reactive protein point-of-care testing may interact similarly with the conceptions and behaviors of patients and health-care providers, but we know little about any such social consequences.

Our objective is to contribute to the social understanding of ABR and its proposed remedies through a comparative qualitative study of fever patients’ health perceptions in the LMIC settings of Chiang Rai (Thailand) and Yangon (Myanmar), where CRP POCT has been trialed. Drawing on themes from the medical anthropology and qualitative clinical research literature, our analysis indicates that local conceptions of illness and medicine do interact with CRP POCT and entail distinctive interpretations, reproduce existing patterns of health-care exclusion, and could lead to potentially risky treatment-seeking behaviors.

## LITERATURE AND FRAMEWORK

### Related literature.

By studying the social context of AMR and CRP POCT, we draw, especially, on the medical anthropology literature. This body of work shares an interest in the varied meanings and manifestations of medicine use, which can have implications for the development of and efforts to address AMR.^6^ For example, a common theme in the medical anthropological literature on AMR is that patients’ conceptions of illness and medicines contribute to locally idiosyncratic patterns of antibiotic use as people use medicines to treat symptoms rather than diseases.^17^

The symbolic meaning surrounding medicine can further influence patterns of use within a society when physical attributes (e.g., color, taste, appearance, and mode of application) are interpreted across cultural contexts.^18^ More generally, the medical anthropology literature suggests that local conceptions are an important driver in medicine use and health behavior and consequently warrant study for antibiotic use in particular.

By virtue of being locally specific, conceptions of medicine and illness—and their implications for AMR-relevant health behavior—vary across social contexts. As our study takes place across two LMIC settings, we also speak to comparative qualitative social research on health-care–seeking behavior. Comparative social research in health is not uncommon, but broader knowledge about the cross-contextual variations of local conceptions and health-care–seeking patterns in the context of AMR could help to situate the usefulness and effectiveness of remedial action and interventions across (cultural) borders.^19–22^ Therefore, our first research question contributes to the social understanding of AMR through a comparative study of Yangon and Chiang Rai: *How do local conceptions of illness and medicine influence context-specific health-care–seeking patterns in Chiang Rai and Yangon?*

Our second research question speaks to the limited social understanding of CRP POCT. The existing literature explores, primarily questions of clinical efficacy through quantitative research, thus characterizing CRP POCT as effective in reducing unnecessary antibiotic prescription in high-income contexts.^10,23–25^ Despite the growing interest in CRP POCT to reduce over-prescription of antibiotics and to slow the development of ABR, qualitative research has lagged behind its quantitative counterpart.^26^ The existing qualitative literature on CRP POCT is limited to high-income contexts and has a clinical focus with an interest in the acceptability of the CRP POCT by studying users’ and patients’ attitudes and perceptions.^27–32^ Contrary to what the medical anthropological literature suggests, this body of work does not address more fundamental social questions relating to the uses and meanings surrounding antibiotic use, which may interact and interfere with the introduction of a point-of-care test. Our second research question corresponds to this social research gap: *How do local conceptions of illness and medicines interact with CRP POCT in primary care settings?*

### Analytical framework.

**Figure 1.**
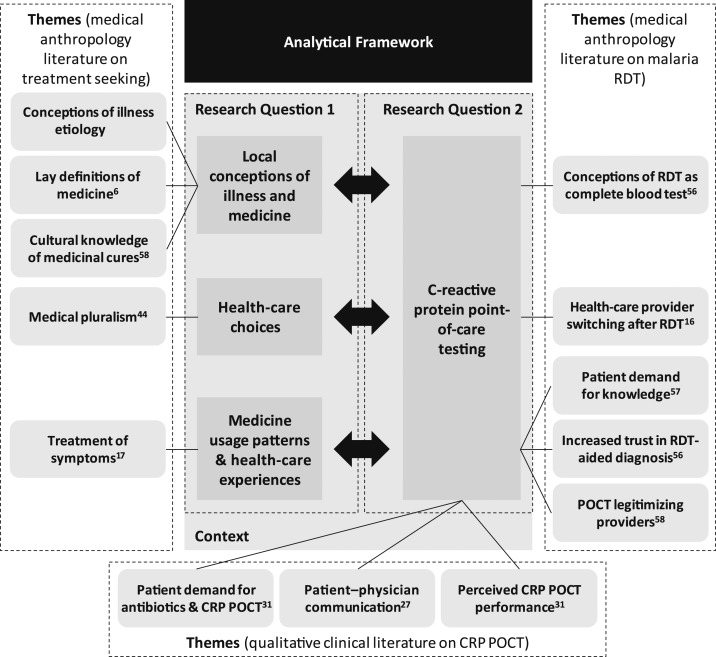
Analytical framework and themes for analysis developed from three bodies of literature.

We structured our analysis according to themes derived from medical anthropology and qualitative clinical research on CRP POCT (summarized in Figure 1. Our analytical strategy for Research Question 1 explored how local conceptions of illness and medicines influence health-care–seeking behavior (e.g., self-diagnosis and treatment choices) and the resulting patterns of access to health care and medicine from formal and informal health-care providers (under the heading “analytical framework” in Figure 1). Specific themes from the anthropological literature, for example, lay definitions of medicine, formed our initial analytical categories.^6^ Analyzing and juxtaposing these analytical domains for Chiang Rai and Yangon thereby helped us to address the lack of comparative qualitative evidence on AMR-related conceptions of illness and behavior. Our analytical strategy for Research Question 2 involved examining the relationship between CRP POCT and the salient themes of Research Question 1, for example, the interaction between CRP POCT and local conceptions of illness. We also related this analysis to themes in the qualitative CRP POCT literature, the limited breadth of which required us to incorporate complementary themes from the medical anthropology literature on malaria RDT. In short, our analysis commenced with 1) understanding the case contexts, followed by 2) examining local conceptions and treatment-seeking behavior among patients in our field sites, and 3) situating the CRP POCT within these conceptions and behaviors to detect potentially unintended interactions and interferences.

## MATERIALS AND METHODS

### Study design.

We conducted cross-sectional qualitative social research that complemented a clinical trial on the introduction of CRP POCT in primary health services in the outskirts of Yangon and Chiang Rai between July 2016 and July 2017. Both sites are AMR priority areas, having reported specific forms of resistance (e.g., scrub typhus in Chiang Rai, *Mycobacterium tuberculosis* isolates in Yangon) and exhibiting a high risk of ABR through human antibiotic use.^33–36^ The clinical trial focused on antibiotic prescription behavior of HCWs (i.e., medical doctors in Yangon and nurses in Chiang Rai). The trial involved 1,200 febrile patients (600 adults/children) per country, who were recruited at three clinics (operated by a nongovernmental organization [NGO]) and one public primary care hospital in Yangon, and at six primary care units in Chiang Rai. The patients were randomized into two treatment groups with different CRP cutoff levels to guide antibiotic prescription, and one control group. At their first visit, the treatment groups watched a short educational video about CRP POCT and the lacking necessity to take antibiotics for viral infections,† received the 5-minute CRP POCT, and carried out a urine test (control groups provided urine samples, venous blood samples, and nasopharyngeal swabs). Study nurses/doctors conducted these activities and presented the CRP POCT results to the HCWs to inform their prescription decisions. All trial participants were shown posters and received information during the consent process to acquaint them with CRP POCT, all received the CRP POCT at their second visit after 5 days to validate the HCW’s treatment decision, and all had a second follow-up visit at Day 14 with questions on recovery.

The social research study complemented the clinical trial to understand the social context of CRP POCT implementation. As we applied anthropological concepts, an ethnographic research design would have been ideal.^37^ However, time constraints imposed by the parallel clinical trial and the probable influence of participant observation methods on the study setting prevented such a design. We, therefore, conducted a cross-sectional qualitative study (designed and led by a social scientist, viz. M. J. H.), which studied the test introduction at the interface between patients and health systems and which drew on theoretical strands from medical anthropology (treatment-seeking behavior), political sciences (street-level bureaucracy), and sociology (actor-network theory). We present the overarching findings of the social study elsewhere and report in the present article a substudy that analyzes in detail the relationships between CRP POCT and patients’ treatment-seeking behavior across two implementation contexts.

### Data collection and analysis.

Semistructured interviews (SSIs) and focus group discussions (FGDs), with a total of 92 participants, including febrile patients and HCWs, were conducted leading to 84:35 hours of recorded material and 936,000 words of original-language transcriptions, translations, and field notes. This article focuses on the patient data from 37 participants from Chiang Rai (25 SSIs and three FGDs) and 21 participants from Yangon (11 SSIs, two FGDs; summarized in Table 1).‡ This patient sample involved participants of both the treatment and the control groups of the clinical trial, plus a group of nonintervention fever patients who had not participated in the clinical study (whom we sampled through separate logs kept at the health centers). The volume of this data corresponds to 44 hours of recorded and 494,000 words of written material. The patients were sampled purposefully for maximum variation across the dimensions of age, gender, education, study group, and antibiotic prescription.

**Table 1:** Sample summary of interview and focus group participants in Chiang Rai and Yangon

		a) Semi-structured interview (SSI) participants			b) Focus group discussion (FGD) participants		
		Yangon	Chiang Rai	Total SSI participants	Yangon	Chiang Rai	Total FGD participants
Age (years)	18–49	9	21	30	6	7	13
	50+	3	6	9	3	3	6
Sex	Female	8	17	25	5	7	12
	Male	4	10	14	4	3	7
Education	≤ 6th grade	3	17	20	1	3	4
	> 6th grade	9	10	19	4	7	11
Study group	CRP POCT treatment	6	13	19	5	4	9
	CRP POCT control	2	8	10	3	3	6
	Nonintervention	4	6	10	1	3	4
Received antibiotic during treatment	Yes	2	13	15	4	6	10
	No	6	14	20	3	4	7
Grand total		12	27	39	9	10	19

CRP = C-reactive protein; POCT = point-of-care testing.

Our thematic analysis builds on the preformulated themes described in Figure 1 and on new themes that emerged from our qualitative data.^38^ The thematic coding was iterative, using NVivo11 (QSR International Pty Ltd., Melbourne, Australia).^39^ Translation ambiguities were minimal as our research team included Burmese and Thai native speakers. We first analyzed the data set based on local conceptions of illness to explore the landscape of meaning within which patients act. Second, we traced popular terminologies that patients used to describe these conceptions, which helped illustrate how conceptions and health-care–seeking patterns are linked. Last, we explored how these conceptions and health-seeking patterns could relate to CRP POCT and reasons why some patients may not receive the test. Our comparative analysis further considered the nature and emergence of themes and sub-themes across and within the case contexts.

## RESULTS

### Case study context.

Chiang Rai is a northern Thai province with 1.1 million inhabitants in 2000, 12.5% of whom belong to minority groups, including hill tribes.^40^ Thailand achieved Universal Health Coverage (UHC) in 2002 and met all health-related targets of the millennium development goals by the early 2000s.^41^ Its geographically dispersed health infrastructure allows near-universal access to public primary health centers and district hospitals.^42^ In addition, Thailand carries out antibiotic stewardship activities through campaigns such as the “Antibiotic Smart Use” (ASU) program aimed at “promoting rational use of medicines” by introducing a set of interventions on individual (e.g., prescribing herbal medicine for nonbacterial infections), network (e.g., sharing lessons learnt), and policy levels (e.g., implementing ASU-related policies at hospitals).^43,44^

In comparison, Yangon, Myanmar’s ex-capital and its most populous city with 7.4 million inhabitants (Department of Population, 2015), has a less developed health system. Health care is provided through both the private and public sectors, but financed predominantly by out of pocket expenditure (50.7% of total expenditure on health as compared with 11.9% in Thailand in 2014).^45,46^ A national health plan (2017–2021) was recently published with the goal of achieving UHC by 2030 (Ministry of Health and Sports, 2017).^47^ Yet, decades of neglect of the health sector have left the Burmese health system behind its Southeast Asian counterparts, visible, for example, in low levels of health expenditure per capita of US$20 in 2014 (Thailand: US$228, Malaysia: US$456, and Vietnam: US$142).^46^ The unstructured nature of the squatter settlements and the emergence of private and unregulated health-care providers within the Yangon study site make the landscape yet more obscure and difficult to navigate. In addition, there is little stewardship from the government in terms of addressing ABR, with no existing national antibiotic-related policy to date.

**Figure 2.**
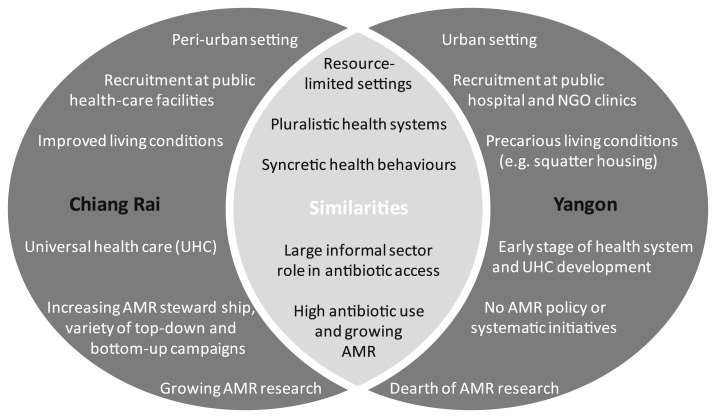
Summary of case context similarities and variations.

Overall, antibiotic access and use is high in Chiang Rai and Yangon, with a large contribution from the private and informal sectors.^48,49^ Differences between the settings emerge in terms of health system development and socioeconomic conditions, where the Chiang Rai site shows improved living conditions, more inclusive health-care access, and a higher level of antibiotic stewardship. Recent data from primary care facilities thereby indicate a decrease of antibiotic prescriptions to outpatients from 30% to 12% in Thailand (2010–2015) and an increase from 38% to 47% in Myanmar.^50^ Figure 2 summarizes the similarities and differences between the sites.

### Research question 1: local conceptions of illness and medicines.

**Figure 3.**
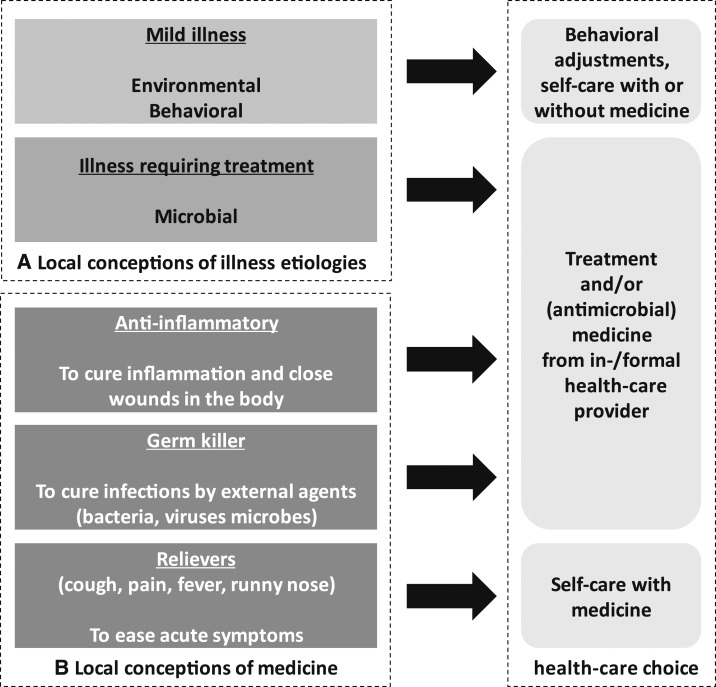
Link between local conceptions of illness etiology, local conceptions of medicine, and health-care choices for fevers and associated symptoms.

As might be expected, we observed a link between self-perceived severity and health-care–seeking behavior.^51^ However, local conceptions of illness etiologies emerged as important correlates because they were intrinsically linked to patients’ symptoms and their self-assessed severity (illustrated in Figure 3A). The three broad categories of illness etiologies described by patients in both settings were environmental (e.g., illness caused by the weather), behavioral (e.g., working too hard), and microbial (e.g., unspecified “*germs*” and “*microbes*”).

Patients typically linked fevers and mild associated symptoms such as headaches to environmental and behavioral causes, whereas they related illnesses with a suspected microbial origin to symptoms that require treatment (e.g., sore throats).§ The ensuing health-care choices were linked systematically to this assessment. For example, where patients considered a fever to have environmental or behavioral causes, their remedial health action would include changing the behaviors that allegedly caused the fever, or self-treatment using “*paracetamol*” and other “*fever reliever*” medicine. Behavioral adjustments were for instance reported by a male patient in Yangon, who explained that he would not buy and use medicine during a fever that was caused by “*lack of sleep*” and instead “*take some rest in bed, in hopes that I will get better*.” In contrast, patients linked more serious symptoms such as sore throats or fevers with multiple symptoms (e.g., pain) to external disease-causing agents such as germs, microbes, viruses, or worms. The subsequent health-care choices would reportedly involve medicines specifically “*to kill germs*” or “*to stop* [germs] *from spreading*” and thus “*protect the body*.” For instance, one Yangon respondent indicated that, “*I think that fever also has germs so there would be germ killer* [i.e., antibiotic] *for fever*.”

Parallel to (and partially overlapping with) local conceptions of illness etiology, lay definitions of medicine deviated from clinical definitions and appeared to exert an influence on patients’ health-care–seeking behavior as well (Figure 3B). For example, in Yangon, the most frequently used terms to describe medicines to treat fever and related symptoms were “*microbe/germ killer*,” “*fever reliever*,” “*pain reliever*,” and unspecified cocktails of “*mixed medicine*.” Among these, the term 
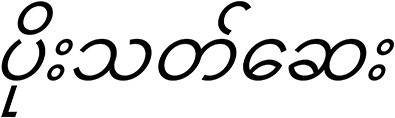
 (*poe thet say*) was the common colloquial reference to antibiotics among both lay people and clinicians, directly translating into “*microbe killer*” or “*germ killer*.” “*Germs*” in this case can refer to a broad range of agents, which maybe bacterial and nonbacterial (e.g., viruses or pests). In contrast, fever patients in Chiang Rai described their medicines with a broader range of terms, including “*anti-inflammatory medicine*,” “*germ killer*,” “*cough medicines*,” “*fever reliever*,” “*pain reliever*,” and “*stomach ache medicine*.” The terms 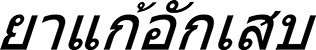 (*yah kae ak seb* or “*anti-inflammatory medicine*”) and occasionally also 
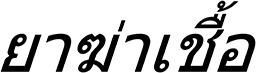
 (*yah kah chuea* or “*germ killer*”) were colloquially used to refer to antibiotics.

The intuition underlying these local definitions can potentially invite antibiotic over-use for conditions with non-bacterial causes. For example, in Chiang Rai, sore throat was described as “*a wound in the throat that is constantly wet*” or as a “*red throat, inflamed throat*” that would be treated thus with “*anti-inflammatory medicine*”—that is, antibiotics. In Yangon, the term “*germ killer*” (i.e., antibiotics) was commonly linked to germs that cause fever, flu, sore throat, rashes, and other conditions such as arthritis. The wide range of potential uses for antibiotics could be related to the term 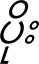 (*poe* or “*germ*”), which includes microbial agents such as viruses and bacteria alongside pests and insects. The term “*germ killer*” can, therefore, refer to pesticides and to antibiotics. In short, where the implied logic of vernacular descriptions of medicine deviates from their clinical definition (and our interviews suggest that this is the rule rather than an exception), patients might access antibiotics for an unexpectedly wide range of symptoms and uses.

Patients, therefore, appeared to be more likely to take antibiotics knowingly or unknowingly when their conceptualization of antibiotics aligned with the perceived nature of their illness (be it fever or other acute conditions), or when it was deemed to have microbial causes. However, time and resource constraints in both sites often meant that these patients accessed medicine through the informal sector, and formal providers, typically, only if private treatment options were unaffordable or if symptoms persisted or worsened. The first resort of care might thus potentially involve unsupervised antimicrobial use, accessed through the informal sector. When formal health-care access actually did take place, patients in both sites expressed that they “*want to feel better quickly*” and “*trust*” the public and private HCWs, but also that they wished to know more about their health but did not receive this information.

Our qualitative analysis, therefore, suggests that our Chiang Rai and Yangon sites shared a common underlying pattern in which patients’ local conceptions of illness etiologies and antibiotics were linked directly and indirectly to health-care–seeking behavior, including behavioral adjustments, self-treatment, and in-/formal provider access, together with expectations of antibiotics for symptoms and conditions beyond their clinical definition. The manifestations of these conceptions varied with the local health systems and provider landscapes across our field sites, but we describe in the following section that they have common implications for the introduction of CRP POCT to guide primary-care-level antibiotic prescription.

### Research question 2: relationship between CRP POCT and local conceptions.

**Figure 4.**
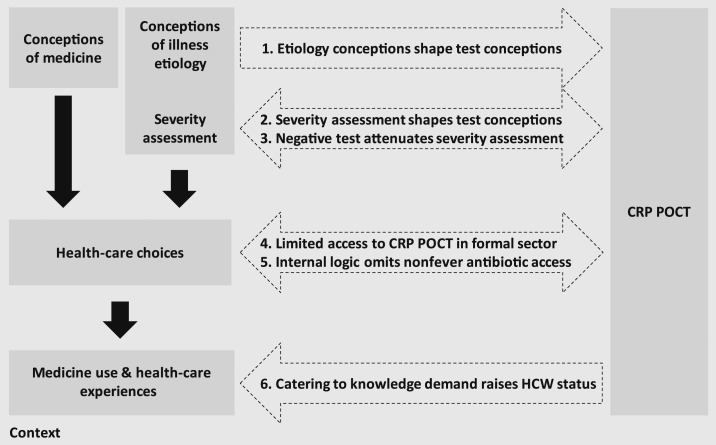
Interactions between local conceptions, treatment-seeking behavior, and C-reactive protein point-of-care testing (CRP POCT).

The introduction of CRP POCT in Chiang Rai and Yangon interacted with patients’ conceptions of illness etiologies, with their assessment of how severe the illness is, with the ensuing health-care choices and, last, with their health-care experiences in the formal health-care sector (summarized in Figure 4). These interactions influence patients’ conceptions of CRP POCT (Interaction 1 in Figure 4), severity assessments (Interactions 2 and 3), exclusion of specific patients and symptoms from CRP POCT (Interaction 4 and 5), and the perception of the primary care facilities who provide CRP POCT (Interaction 6). Although these interactions emerged consistently across our two field sites, patients’ education contributed to additional within-site variation.

A range of patients had a vague notion that CRP POCT related to medicine prescription, but four distinct conceptions about the test emerged among our respondents:

Conception 0: Patient does not know purpose of the test (common)
Conception 1: A test that distinguishes bacterial from nonbacterial causes of infection (rare)‖
Conception 2: A test for specific diseases that patients typically deem “*serious*,” such as syphilis, diabetes, dengue, tuberculosis (TB), or human immunodeficiency virus (common)
Conception 3: A comprehensive blood test to detect any disease in the human body (common)¶

Although all these conceptions emerged in both field sites, they varied across patients’ education levels. This relationship was not straightforward, however. Chiang Rai patients with primary education or below had a wide range of interpretations of CRP POCT (e.g., Conceptions 2 and 3), whereas patients with secondary and higher education in Yangon related to Conception 1 and to conceptions that are at odds with CRP POCT’s clinical intentions (e.g., Conception 2). These emerging patterns suggest that CRP POCT inclusion and adherence may have social gradients within one implementation setting.

Beyond education, conceptions of microbial illness etiologies and their severity assessment appeared to influence interpretations of CRP POCT as a test for specific diseases (Conception 2) or as a comprehensive blood test (Conception 3). C-reactive protein point-of-care testing in our case studies was provided in formal primary care facilities, to which patients often resorted for illnesses with persistent symptoms or that cater to “*serious*” infections potentially caused by “*germs*.” Patients, therefore, appeared to associate the test with serious specific conditions (Conception 2). This may have also influenced the interpretation as a test to detect microbes in the body (Conception 3), considering that “*microbes*” did not only include bacteria but also germs, worms, or pests in the local vernacular, and that patients in Chiang Rai and Yangon did not necessarily differentiate between these explicitly. Consequently, patients in both sites were “*relieved*” or “*happy*” when tested negative, for example, a female respondent from Yangon receiving the CRP POCT stated that “*When I got the results from the doctor, he said that it’s all good, so I am happy, just scared that they might find something in the blood*.” We see this as evidence of a plausible influence of local conceptions of illness on the interpretation of the CRP POCT beyond its clinical intentions (Interaction 1, Figure 4), of an association of the test with more severe conditions that require treatment in formal health-care facilities (Interaction 2) and, conversely, an effect of the test in reassuring patients that they did not have a serious illness (Interaction 3).

Furthermore, CRP POCT interacts with people’s treatment choices as 1) the delivering health-care facilities are not equally available for all patients (Interaction 4) and 2) the logic of distinguishing bacterial from nonbacterial fevers (or respiratory infections) can exclude a potentially wide range of antibiotic-seeking behaviors for other symptoms within and beyond the formal health-care setting (Interaction 5). Patients in both field sites lamented access constraints to the formal health-care services that provide CRP POCT. For instance, an older female respondent in Chiang Rai postponed access to the health center for 2 weeks because “*there was no one* [in her household] *to take me there*,” and respondents in Yangon alluded to long travel times and high costs to access the CRP POCT facilities, because of which “*there are times where we miss the appointments*.” Resource and time constraints may, therefore, bias formal health-care utilization toward priority members of the family—especially children. Patients who may only seek informal health services for mild illnesses would then be less likely to receive CRP POCT, which appeared to be a common case in Yangon’s more fragmented health-care system where unlabeled medicine cocktails (“*mixed medicine*”) from a nearby drug shop would cost as little as 3% as a journey to a free-of-charge clinic participating in the CRP POCT trial according to our respondents. Yet, even if patients do access formal health-care services, the internal logic of the CRP POCT may exclude a range of patients with expectations to receive antibiotics for illnesses outside of the scope of the test. It was for instance common in Chiang Rai that patients expected “*anti-inflammatory medicine*” (i.e., antibiotics) for sore throats but not for fevers (the focal inclusion criterion of the CRP POCT trial), whereas other patients reported that they had received antibiotics at health centers because of “*bumps from mosquito bites*” and “*when it’s bad. If I have muscle pain*” (for which they would not be CRP tested).

Last, among patients who received CRP POCT, almost all had favorable opinions of the test and stated that they trusted the HCW’s diagnosis more with the test. Yet, these patients did not favor the test because of its intrinsic purpose but rather because they received more time, attention, and explanations from HCWs (a similar pattern emerged among the control group of the study, from whom a venous blood sample was taken). For instance, good treatment was often related to “*a careful checkup*,” whereas clinics with poor treatment “*don’t have enough time* … *and then send us off* (… without giving us) *time to ask* (questions about what is wrong with us).” Because CRP POCT intervention typically added a set of extra procedures and 5–10 minutes to total consultation time according to our observations in the field, respondents in the intervention groups considered that treatment is “*better with the blood test*. *They* (HCWs) *take care of you*” and stated explicitly that “*I like that they* (the hospital doing CRP POCT) *check the urine and the blood*.” Treatment decisions based on CRP POCT results were also deemed more “*precise*” and “*sophisticated*,” especially in Chiang Rai where fewer patients had previously experienced point-of-care testing at primary care facilities (contrary to the NGO clinics in Yangon specializing in TB and sexually transmitted diseases). These impressions suggest that CRP POCT elevated the status of primary care HCWs without necessarily leading patients to appreciate its intrinsic nature, given that patients consistently developed different interpretations of the test in line with their preexisting conceptions of illness (Interaction 6).

## DISCUSSION

### Limitations.

Before we continue with discussing the implications of our study, it is important to highlight research limitations related to sampling and researcher positionality. First, our sample cannot represent the Chiang Rai and Yangon populations because it comprised fever patients with formal primary health-care access. Our findings are, therefore, prone to misrepresenting subpopulations who would normally seek antibiotic treatment of other symptoms or diseases such as muscle pain or urinary tract infections, or who do not access primary health services either by choice or other constraints (e.g., poverty). We used a purposive maximum variance sampling strategy to include a diverse patient pool and mitigate the impact of patient misrepresentation.^52^ In addition, our interviews included questions on the health-care provider landscape, health-care choices, and other illness episodes of our respondents and their peers beyond fever and primary health service utilization. This is only a partial remedy. Our study, however, enables a first glimpse into how AMR-relevant conceptions of illness and medicine may compare across neighboring LMIC contexts, and how they interact or interfere with external interventions such as CRP POCT. Further research would broaden our understanding of the nuances and common patterns in people’s antibiotic-related conceptions and behaviors.

The research also faces positionality challenges as different researchers participated in different stages of the research process.^53^ For instance, the overarching clinical study was led by a health economist in collaboration with medical researchers; the social sciences substudy was headed by a European social scientist with a background in development studies, who was assisted by central Thai and Yangon Burmese research assistants. This structure often led the social research team to be perceived as medical researchers despite explicit and repeated assurances to the patients that we were not medical doctors. More generally, the involvement of various researchers with their own preconceptions and positions bears the risk of distorting patients’ voices. Our approach to ensuring an authentic and trustworthy account of our informants’ narratives involved regular team discussions and reflexive exercises to understand each members’ positions and the challenges they faced during the research process, and triangulating of the data analysis across researchers and data types (field notes, SSIs, and FGDs).^54^ However, these remedial actions cannot rule out a residual bias introduced through our positions as outsiders from our specific study communities.

### Relation to the literature.

Our research reinforces existing themes in the general and RDT-specific medical anthropology literature, whereas also hypothesizing new mechanisms underlying the emergence of potentially unforeseen interpretations and implications of medical interventions such as CRP POCT. For example, our comparative analysis echoes medical anthropology themes such as the symbolism and lay definitions of medicine.^6,18,55^ To an extent, our study also reflects themes from the malaria RDT literature, such as conceptions of POCT as a comprehensive blood test to detect illnesses and the satisfaction of patient demand for knowledge and elevated trust in HCWs when a test is used.^56–59^

Yet, our study also adds to knowledge by identifying local conceptions of illness etiologies and patients’ self-assessment of their illness severity as critical mechanisms leading to unforeseen interpretations and consequences of CRP POCT. Spanning varied conceptions of CRP POCT; behavioral implications of negative testing; exclusion from the test for specific target groups, symptoms, and health-care choices; and the satisfaction of health-care demands without appreciating the intervention for its intrinsic purposes, we have established an arrangement of social consequences of CRP POCT that forms a coherent yet deviant system of conceptions and action. C-reactive protein point-of-care testing is an active element of this system that contributes to health-care–seeking practices and new forms of health-care inclusion and exclusion. Considering that these implications materialize consistently across two LMIC case contexts, we see scope in applying our framework to other social research studies aiming to understand interactions between preexisting local conceptions and medical interventions.

### Implications for CRP POCT.

C-reactive protein point-of-care testing may have different consequences for patients who were able to access the test and patients who were excluded. Included patients could potentially benefit from the direct purpose of the test by not receiving antibiotics unnecessarily for non-bacterial infections. The satisfaction of knowledge demands (through receiving test results) and higher health-care satisfaction (through more time, attention, and procedures devoted to patients) could also stimulate increased formal health-care utilization and, therefore, less risky health behaviors for this group. However, not all patients included into CRP POCT would realize these benefits. For example, less educated people in Chiang Rai and more educated people in Yangon had more varied conceptions about CRP POCT. Some of these conceptions considered CRP POCT as a test for specific diseases such as diabetes or as a general comprehensive blood test to detect any disease in humans. Where this is the case, patients may potentially interpret negative test results as “*everything is normal*” and being free of disease, and adjust their health behavior accordingly (this would not be problematic for a common cold, but consider e.g., a hypothetical diabetic patient believing he or she is free of diabetes after a negative test result).

Although it would not entirely resolve risky behaviors and unforeseen interpretations of CRP POCT, a partial remedy may be to emphasize HCW-to-patient communication during HCW’s CRP POCT training, drawing on local notions of illness when explaining the test to patients and asking the patients to re-explain the purpose of the test. The critical reader may observe that such a suggestion cannot be derived solely from patient perspectives, considering for instance the extensive anthropological research on malaria RDT, which pointed at numerous factors including experience with and availability of other diagnostic tests, expectations of positive test results, or time constraints when HCWs make and communicate POCT-based prescription decisions.^14,15^ We acknowledge this limitation and note that our own research with HCWs in Yangon and Chiang Rai (reported elsewhere, using a street-level bureaucracy approach to study behavior within the context of pressure from guidelines and patient demands) indicated a vast range of antibiotic prescription and nonprescription behavior, and HCWs in both settings used colloquial (e.g., “*germ killer*” in Yangon) and technical terms when referring to antibiotics. This suggests that HCWs may share the same interpretation and world views of the community although also facing constraints from policy and patients that could render lengthy communication with the patient infeasible.# Although our analysis of patient perspective, therefore, suggests that the articulation and explanation of conceptions of CRP POCT during the consultation could aid with reducing anxiety and risky behaviors, there is little doubt that such an approach would require further ethnographic research of the patient–HCW encounter in the context of CRP POCT.

Problematic might also be the implications for patients who are excluded from CRP POCT. On the one hand, CRP POCT might simply mirror other interventions that align with the “inverse care law,” adding to a portfolio of formal primary care services that are inaccessible to marginalized populations.^60^ On the other hand, a distinctive form of exclusion may arise from the mismatch between local conceptions of illnesses requiring antibiotic treatment, and the diagnostic focus of CRP POCT. In the case of the clinical trial alongside which our study took place, the focus on febrile patients may have excluded those who expected antibiotic treatment of a sore throat or muscle pain at the same health-care facility. Should CRP POCT become a standard procedure in the future, a possible avenue worth exploring may, therefore, be to test *after* clinical examination if this indicates that antibiotics are needed. Although this solution would require clinical validation of the CRP POCT for a wider range of health conditions, it could potentially avoid a mismatch with local expectations for primary-care-level antibiotic treatment in the absence of bacterial infections, for example, for muscle pains.

## CONCLUSION

Antimicrobial resistance is a global health issue, and point-of-care biomarker tests are among the portfolio of proposed solutions to address the over-prescription of antibiotics for diseases without bacterial causes. This article aimed to expand our understanding of the social context of CRP POCT testing in LMICs. We have built an analytical framework based on existing themes in the medical anthropology and qualitative clinical research literature, and applied it successfully to a unique qualitative data set collected alongside the introduction of CRP POCT at formal primary health-care facilities in Chiang Rai (Thailand) and Yangon (Myanmar). We find that local conceptions of illness are the foundation from which a wide range of interpretations of CRP POCT, distinctive patterns of exclusion, and new treatment-seeking behaviors emerge. Although our study suggests that future CRP POCT interventions may benefit from reconfiguring the test as a validation tool for treatment decisions (rather than as a diagnostic aide for fevers or respiratory infections), we concede that our study has only been one of the first steps toward a better social understanding of clinical interventions to address antibiotic use. We, therefore, hope that the lessons from this enquiry will not only inform clinical research and the implementation of POCT solutions in LMIC contexts, but also stimulate more comparative social research against the backdrop of AMR.

## Footnotes

† The video is available at https://youtu.be/hJnxSwbqWOE.

‡ The Yangon data are lesser in volume because it was collected specifically for a comparative analysis.

§ Note that we are not implying that patients assessed different causes of illnesses as differently severe. Although they were systematically linked to severity and health-care choices, our interviews suggest that the assessment was based on symptoms and other factors such as disease duration, rather than their notions of etiology. Note that “symptoms requiring treatment” are not limited to microbial origins.

‖ This category only comprised patients in the treatment groups who mentioned occasionally the terms “*bacteria*” and “*virus*” and who saw educational video material about the CRP POCT. However, the patients’ narratives suggest that the terms entered their vocabulary and connected and resonated with existing notions gained from the media, but they did not integrate into existing conceptions of illness and remained passive thus.

¶ Although it might be expected, Conceptions 2 and 3 were not exclusive to patients who actually received an intravenous blood test in addition to CRP POCT (but who typically did not learn the results thereof).

# We thank an anonymous reviewer for raising this point.
